# Online Distance Education and Transition to Parenthood Among Female University Students in Sweden

**DOI:** 10.1007/s10680-018-9503-3

**Published:** 2018-11-28

**Authors:** Linus Andersson

**Affiliations:** Swedish Institute for Social Research, Universitetsvägen 10 F, Stockholm, Sweden

**Keywords:** Technology, Fertility, Sweden, Education, Student fertility, Fertility postponement

## Abstract

The expansion of tertiary education is key to understanding postponement of first births. Currently, online distance education is changing the nature of university enrolment. In this study, I suggest that online distance education impacts on fertility by facilitating the transition to parenthood among students. I examine the relationship between online distance education and first births during university enrolment. Using survival analysis of register data for the 1968–1991 female cohorts, I examine the impact of distance and campus education on first-parity transitions during university enrolment between 2004 and 2012 (*N* = 938,768). Results indicate that the negative association between enrolment and first parity conception differs substantially between campus and distance enrolment. Compared to non-enrolment, the hazard of first parity conception is 70% lower during campus enrolment but 43% lower during distance enrolment. These findings are discussed in relation to educational heterogeneity and fertility postponement and the impact of technological innovation on family dynamics.

## Introduction

University students have low rates of first birth, despite favourable age and sex ratios on campus (Begall and Mills 2012; Blossfeld and Huinink 1991; Hoem, 1986; Lappegård and Rønsen 2005; Baizán and Martin-Garcia 2006; Tesching 2012; Thalberg 2013). Increased enrolment in higher education is a key component in the postponement of parenthood (Balbo et al. 2013; Blossfeld and Huinink 1991; Skirbekk 2008). Today, student populations are still growing or have stabilized at high levels. Moreover, tertiary enrolment itself is increasingly being postponed to older ages (OECD 2009), which is likely to further increase postponement. Importantly, the completion of education is central to age at first birth (Ní Bhrolcháin and Beaujouan 2012; Neels et al. 2017). Consequently, factors that affect the timing of fertility *after* the completion of tertiary education can necessarily only explain a limited amount of the variation in age at first birth. It is therefore essential to understand which factors influence the transition to parenthood *during* tertiary enrolment.

One such factor may be online distance education (ODE). Parallel to the trend towards expansion in higher education, the last decade has seen an even more rapid growth in ODE (Allen and Seaman 2010). Universities are increasingly offering distance courses and full programmes at distance, and some are exclusively using ODE platforms (Cowen and Tabarrok 2014). In Sweden, up to 20% of enrolled students in 2009 obtained credits from courses conducted at a distance (Amneus et al. 2011). ODE platforms offer flexibility in combining studies, work and activities in the home (Edmonds 2010; Mårald and Westerberg 2006). The next section will therefore argue that ODE may facilitate childbearing and childrearing among students.

The central role of higher education for postponed fertility is well documented (Blossfeld and Huinink 1991). An expanding literature has also documented that the transition to parenthood differs substantively between study disciplines (Hoem et al. 2006), study intensity (Spéder and Bartus 2017) and occupations (Begall and Mills 2012). The teaching platform (ODE vs. brick-and-mortar enrolment) represents an unexplored part of this puzzle. Fertility researchers have emphasized that changes in the flexibility and organization of educational institutions are central to understanding and formulating policy regarding the timing of first births (Lutz and Skirbekk 2005). Policy advocates have also argued that distance tertiary education is necessary to meet the needs of students with increasingly heterogeneous life courses (Peters 2009). To date, however, there is no empirical research on the association between ODE and the transition to parenthood. Furthermore, the association between ODE and student fertility constitutes a concrete example of how technology may impact on fertility (e.g. Bellou 2015; Chesley and Johnson 2014).

Students may select into ODE on the basis of childbearing intentions, and ODE may stimulate fertility among students. Hence, new technologies may be producing a horizontal differentiation of higher education based on student childbearing behaviour. The present study focuses on establishing an associative relationship by asking: Are university students more likely to become parents during online enrolment than campus enrolment?

I use individual level longitudinal data obtained from national registers, which include the entire female population of Sweden in the cohorts born between 1968 and 1991. Uniquely, Swedish educational registers also include data on the teaching platform (campus or distance) for each academic course. For each academic term between 2004 and 2012, I distinguish between states of enrolment pursued with or without the inclusion of a substantial amount of distance studies. Controlling for study types (full- or part-time studies, degree completion and continuity) and sociodemographic characteristics, I model the risk of first parity conception during these forms of enrolment, compared to non-enrolment, using event-history models.

## Theoretical Framework and Previous Research

Below, I briefly discuss the theoretical explanations and known correlates of student parenthood and the role of educational enrolment for the transition to parenthood. I also present an argument about the role of ODE for student parenthood and outline a general hypothesis.

### University Enrolment and Fertility

Educational enrolment and level of educational attainment are critical aspects in most theories on the timing of first birth. According to new home economics (Becker 1981), the decision to start a family takes the form of a cost–benefit calculation, with the costs consisting in time spent on parenting instead of labour or human capital accumulation. Foregoing the latter is more costly for those with a high or prospectively high earning capacity, which produces a lower likelihood of first birth among the highly educated and those enrolled in higher education (Gustafsson 2005). Empirical support for this theory is found in the positive relationship between childlessness and the level of education across many countries and institutional contexts (Wood et al. 2014). However, the most prevalent pattern is that rather than rejecting parenthood altogether, individuals with a tertiary education enter parenthood after tertiary graduation, at a later age than those with lower levels of education, but quite rapidly following the completion of their university studies (Blossfeld and Huinink 1991). Therefore, low fertility among students is commonly construed as reflecting a deliberate postponement behaviour. One important implication of this conclusion is that in addition to the post-enrolment fertility delay found among university graduates (Gustafsson 2005), enrolment itself acts as a central proximate factor behind increased age at first birth (Ní Bhrolcháin and Beaujouan 2012; Blossfeld and Huinink 1991; Hoem 1986).

For students then, the postponement of parenthood is in part a strategic choice to increase the payoff provided by higher education, e.g. in the form of labour market positioning (Sweeney and Cancian 2004) and finding a highly educated partner (Oppenheimer 1994). At the same time, however, there are more rudimentary explanations for fertility postponement among students. Studying requires time and effort (Blossfeld and Huinink 1991). The need to commute or travel locally to attend courses compromises social interactions in the non-university social sphere and complicates childrearing. Labour market attachment, which is often a requirement for becoming a parent, may be hard to achieve in combination with rigid study schedules (Spéder and Bartus 2017). Further, exposure to and expectations of campus culture, including scheduled activities and extracurricular activities, may distract from or be difficult to reconcile with family formation. Physical social interactions with students, who are less likely to have near-future childbearing in mind, may also offset social learning mechanisms (Bernardi 2003) that promote childbearing plans. Age and sequencing norms further steer the transition to parenthood towards the period subsequent to the conclusion of higher education (Blossfeld and Huinink 1991; Liefbroer and Billari 2010).

Previous research on determinants of student fertility has focused on economic activity and part-time work among students. Spéder and Bartus (2017) have noted that working during one’s studies is a predictor of student first birth transitions and they suggest that such “double-status positions” enhance student fertility by mitigating role conflict. In Sweden, Thalberg (2013) has presented similar results and suggests that since earnings are related to eligibility for parental leave insurance, working students will have higher fertility rates. Having contact with the labour market as a student might also provide the means to satisfy the “affordability clause” (Rindfuss and VandenHeuvel 1990) for childbearing, overcoming a critical amount of uncertainty and risk. Part-time students have been found to be more likely than full-time students to marry (Thornton et al. 1995), and this finding could extend to parenthood. A number of studies have also found correlations between student fertility and educational domains leading to occupations that might be easier to combine with childbearing, such as teaching (Hoem et al. 2006; Lappegård and Rønsen 2005; Baizán and Martin-Garcia 2006; van Bavel 2010). In contrast, parent students in regular brick-and-mortar education report a lack of institutional support (Brown and Nichols 2013), that they struggle with schedules that are difficult to reconcile with parental obligations (Moreau 2016) and that they have to cope with stigmatizing stereotypes from the university campus environment, which portray them as both bad students and bad parents (Estes 2011).

To summarize, the impediments to entering parenthood as a student are not only linked to the issue of utility maximization based on earnings capacity, they are also a matter of practicalities. This is an important distinction, since it indicates that students may be willing, but simply not *able*, to have children. It is possible that many students are focused on optimizing their human capital, whereas others may be less concerned about their labour market prospects (Hakim 2003) and might consider becoming a parent during enrolment, provided that it is possible to resolve certain critical practical issues. On the basis of this perspective, we might expect that various factors linked to the study environment, including certain technological and institutional innovations, may predict student fertility.

### Online Distance Enrolment and Fertility

ODE has been promoted for its presumed fit to the needs of non-traditional students (Cowen and Tabarrok 2014). It has been argued that ODE bridges both cultural and physical distance (Jacob et al. 2016) and offers a flexibility that enables individuals to combine studies with adult roles and responsibilities (McIntosh 2005). University students have reported flexibility to be an important reason for choosing an online study platform (Kowalski et al. 2014). In interviews, distance students have emphasized that online platforms help them to combine the parental obligations with higher education (Edmonds 2010). In Sweden, general skill attainment as well as flexibility and overcoming distance barriers have been mentioned as motivations for participating in online education (Mårald and Westerberg 2006). If ODE enables the combination of university studies with other engagements, it influences at least three interconnected mechanisms that impact on student fertility: the cost of childbearing, parental leave eligibility and role conflict. ODE may influence the cost of having a child during the specific life course phase of university studies (Gustafsson 2001, 2005). First, childbearing entails opportunity costs in terms of foregone investment in human capital, such as education and work experience, as well as the direct loss of income from paid work, and direct monetary costs of childrearing. But provided that childlessness is not considered an option, one may find it efficient to have one’s first birth as a student because the opportunity costs of lost income and labour market experience are lower for students (as students normally do not work full-time to begin with). Furthermore, the presence and anticipation of a motherhood wage penalty might make student childbearing particularly appealing to women, since one factor affecting the motherhood wage penalty is the loss of work experience during the child’s infant years. In anticipation of employer preconceptions and discriminatory practices towards employed mothers, the prospect of engaging in careers that are unbroken by lengthy leaves might be highly valued. However, the time costs of studies entailed by transportation and participation at fixed hours need to decrease enough for this parallel activity to be manageable. To the extent that this necessary flexibility is enabled by ODE, distance enrolment may have a positive effect on student childbearing. Second, in welfare states that condition parental leave on individual income, employment can be associated with a rapid transition to childbearing (Matysiak and Vignoli 2008). Students who also work are not locked out from the parental leave system. Eligibility for parental leave receipt might matter for the childbearing decisions of students. Again, if ODE provides the flexibility to maintain a connection to the labour market during studies, this enables the transition to parenthood. Third, it has been proposed that sequencing norms, and norms about the conditions under which a child should be raised, prevent student childbearing (Blossfeld and Huinink 1991). The role of student is arguably tied to the practices involved in participation at the many venues of the university. The role of mother and carer is tied to the household. Because of their ability to separate themselves from the campus environment, distance students may be less subjected, or less exposed, to negative sanctions and expectations tied to their role as students and mothers. If parallel employment also shifts the students towards the normative role of earners (cf. Spéder and Bartus 2017), and ODE accommodates such a combination, then distance enrolment is an enabling factor for student fertility. The mechanisms behind direct effects of ODE on childbearing are intrinsic to indirect effects that predict selection into ODE based on childbearing intentions. The difference lies in when and how childbearing decisions are made. Childbearing plans are formed continuously. ODE may be used strategically to accommodate childbearing, since parenthood is potentially easier to combine with ODE than traditional studies. At the same time, a re-evaluation about childbearing plans and decisions while in distance education, regardless of the initial reason for enrolling, could tilt actions in favour of childbearing.

Previous research clearly shows that flexibility, mobility and being independent of the campus environment are beneficial to student parenthood and that online education is characterized by flexibility, mobility and independence from campus. Hence, ODE may positively influence the perceived and actual feasibility of making the transition to parenthood as a student. Students who are already looking to have a child may select into this platform, while students who choose ODE for other reasons may find the option of becoming parents to be consistent with this form of study. This leads to the study’s main hypothesis: enrolment in distance education will be less negatively associated with transition to parenthood than enrolment in campus education. Of course, the reasoning above also predicts distance enrolment to occur shortly after transitioning to parenthood. This follows from the assumption that individuals formulate childbearing decisions in anticipation of the future. An awareness that studies can be pursued from home after childbirth, via distance studies, would promote both childbearing and subsequent enrolment in ODE. This is the reverse temporal sequence but is indicative of the same substantive trajectory of combined studies and parenthood. In the event-history framework of this study, I focus on analysing the impact of distance enrolment on the risk of transitioning to parenthood, but provide complementary analyses of parity, conception and platform type in “Appendix” Table 10.

## The Swedish Context

The Swedish educational system is characterized by a low degree of tracking and a high degree of tertiary enrolment, free tuition at all levels and universal student loans and entitlements. For higher education, the level of study activity and parallel employment among Swedish university students lies at the average in a European comparison (Eurostudent 2015). It is possible that the subsidized financial situation of students eases the transition to parenthood. At the same time, a generous earnings-related parental leave system encourages stable employment prior to childbearing (see Thalberg 2013 for an extended discussion). In comparison with many other Western countries, the transition to parenthood during and after enrolment might be more compatible with Swedish conditions (Billari and Philipov 2004; Liefbroer and Corijn 1999).

Swedish university students either compile sets of courses freely or study within the framework of a more restrictive programme. As there are only a few fully online distance programmes and no exclusively online universities, by far the most prevalent use of distance education involves alternating between distance courses and on-campus courses as a means of compiling the requisite number of courses to complete tertiary education. Sweden was relatively early in its development of ICT. In 2004, all municipalities had at least one broadband provider (PTS 2004), and about 80% of the population aged 18–44 had access to the Internet at home. By 2010, this figure had increased to over 95% (Finndal 2010). The number of students enrolled in distance education increased by 100% between 2002 and 2009 (Amneus et al. 2011). In 2011, every fourth university student had registered for at least one credit-awarding online distance course (Swedish register data, author’s own calculations). These figures are comparable to those of other developed countries such as the USA (Allen and Seaman 2010). Cross-sectional bivariate measures indicate that online students tend to have lower retention rates, are more likely to be female, and are older than campus students (Amneus et al. 2011).

## Method

### Data and Sample

The paper employs administrative register data from Statistics Sweden covering the entire Swedish population. The data provide longitudinal monthly information on childbirths. The date of conception is defined by subtracting 9 months from the date of childbirth. Information on tertiary enrolment on individual courses is available per term. I include all women born between 1968 and 1991 who were registered as living in Sweden in 2004, giving a total of 1,458,241 individuals. Since the characteristics of distance students are less well known than those of campus students, I did not restrict the sample to typical enrolment ages. The population examined includes all individuals who could have entered tertiary education during the observation period and who could be followed for at least 3 years between the ages of 18 and 44. The results section will present year and cohort-specific regressions. The observation window spans the period from 2004 to 2012. The year 2004 was chosen as the starting date due to the availability of data and because by then the level of Internet access was high and the supply of distance courses was quite extensive. The final observation year, 2012, corresponds to the latest available data. After applying left censoring of conception prior to 2004, the analytical sample consists of 938,768 individuals, of whom 345,232 experienced first parity conception during the observation period.

### Educational Measures

Studies are performed in different ways and student fertility may be limited to sporadic, non-continuous, non-committed forms of education. Previous measures of online education consist mostly of dichotomous indicators, e.g. ever being in education in a given year. This disregards the extent of students’ studies (e.g. Allen and Seaman 2010) and it seems plausible to distinguish between enrolment in substantial amounts of ODE and spells where online studies constitute only a small proportion of all studies. I differentiate between part-time and full-time studies. A term in which a student accumulated at least 22.5 study credits (75% of full-time activity or more) is considered full-time; a term producing fewer study credits is considered as being part-time. The academic terms are set to overlap the summer break so that term one runs from January to June, while the other runs from July to December. Completed rather than registered courses are used for all tertiary categories due to variation in attrition between platforms.

For each academic course, there is information on whether or not it was given at distance. The official criterion for reporting a course as a distance course is that “Teacher and students are separated in time and/or space”. This could include any procedure, ranging from letter correspondence to web-based courses, but distance must be the mode of learning. By 2004, however, it is reasonable to assume that all distance courses were supported by online platforms in some sense, as email correspondence was common practice by this time. The lion’s share of distance education is pursued in alternation with campus education. Since the most prevalent usage of online courses involves taking a mix of online and campus courses during the years of formal education (rather than exclusively online or campus trajectories), the distinction made in the analysis focuses on the amount of distance online platform usage during a given term. A dummy for distance education takes the value one if at least 15 distance course credits were accumulated during the term in question (corresponding to half a term of full-time studies). Thus, full-time terms (75% study activity or higher) and part-time terms^1^ (less than 75% study activity) are both considered to contain *a significant use of distance education* if at least 15 credits (50% or more of full-time study activity) relate to online courses. The selection of threshold levels is discussed in the results section and tested in Table 3. Information on the study platform (online or campus) is only available for higher education. Secondary and non-tertiary post-secondary education is measured using registration information on a yearly basis. This operationalization results in eight discrete time-varying states: non-enrolment (reference category), upper secondary education, post-upper secondary non-tertiary, full-time tertiary with less than 15 ECTS distance credits; full-time tertiary with 15 or more ECTS distance credits, part-time tertiary with less than 15 ECTS distance credits, part-time tertiary with 15 or more ECTS distance credits.

### Other Measures

I include two variables to account for confounding effects of social origin. Parental SES is associated with tempo in the transition to parenthood, and the efficient use of new technologies may be stratified by social origin. I include a proxy for *Social class background*, based on the parent’s occupation [whichever parent has the highest ranking occupation (Erikson 1984)]. Decennial census data from 1960 to 1990 include socio-economic index scales (Erikson and Goldthorpe 1992) that are coded into five levels closely resembling the EGP schema (labour contract, mixed contract, professional, proprietors and farmers and unknown). Taking online courses may place greater demands on language proficiency. For this reason, I include a dummy variable labelled *Migrant background* indicating whether or not an individual was born in Sweden.

Transition speed varies by the level of education attained and this may also be the case for students. Educational level might confound the results if distance users have obtained a degree while enrolled to a greater extent than campus students. Therefore, I include a yearly time-varying, non-lagged, categorical variable for completed *Educational level* (compulsory, secondary, post-upper secondary non-tertiary, tertiary graduate or at least 2 years of studies, tertiary postgraduate.) If sparsely populated and remote areas disproportionately use online education, regional variations in fertility might confound the effects of distance education on student fertility. I therefore include a yearly time-varying dummy indicating the characteristics of the *Region of residence* in which an individual is registered at any given time (metropolitan, urban and rural) from the “H-region” classification of Statistics Sweden (SCB 2015).

Partner resources predict fertility among students and could confound the relationship between platform and event. C*ivil status* is included as a yearly time-varying categorical variable, denoting individuals as married, not married or of unknown status. Unfortunately, information on cohabitation is not available for the present sample. Distance course-takers may on average be older. As age is associated with likelihood of first parity conception, *Age* and *Age squared* are included. Calendar year is included to account for *Period* effects.

### Analytical Strategy

The first conception event is observed from 2004 to 2012. Individuals enter at-risk status from the age of 18 and onwards, depending on cohort entry. Individuals are censored at event, death, migration, turning 45, or in April 2012. I use piece-wise constant baseline intensity models (Hoem 1993). The strategy used here is to utilize the population scale data to show main effects across detailed educational groups and relevant subgroups, paying special attention to practices that may differ between online and campus platforms (e.g. Lappegård and Rønsen 2005).

First, I describe the analytical sample. I show how different proportions of online education within a term are related to student fertility. Second, I examine the general association between transition to parenthood in full- and part-time distance studies and campus studies, relative to non-enrolment and disaggregate this model by age and cohort. Third, I disaggregate the model by disposable income quartiles and educational field. For income measures, the age-specific rank of disposable income was calculated based on the entire Swedish population for each calendar year. While working hours would be a preferable variable, this measure captures available resources and provides a rough indication of work activity. For educational field, I use Statistics Sweden’s schema that identifies the following separate fields: education and teaching, humanities and art, social science, natural science, technology, agriculture, health and services. Finally, I present results from sensitivity analyses, i.e. a set of complementary analyses that tap into the choice of distance versus campus education (Table 8), the transition to second parity (Table 9) and the implication of partner resources (Table 10). These analyses are restricted to “Appendix” as they extend beyond the aim of the present study, but nonetheless provide the reader with important information about ODE and fertility.

All models, unless otherwise specified, employ non-enrolment as the reference category. Results are presented as hazard ratios with 95% confidence intervals. Unless otherwise stated, all models include controls for period, age, age squared, social background, region of residence, migrant status, civil status and educational level.

## Results

### Descriptive Results

The upper section of Table 1 shows the number of individuals, conceptions and person-months by different enrolment statuses. 40,495 individuals can be seen to be in full-time distance enrolment and 24,326 in part-time distance enrolment. 1573 (full-time) and 1157 (part-time) conceptions have been recorded for these groups. Together they account for 9% of the observed tertiary enrolment person-months. The lower section of Table 1 shows the percentage of person-months across all variables used by campus and distance enrolment spells. Note that the data only pertain to the period 2004–2012 and only to women who have not reached first parity. In the present sample, the ages at which distance enrolment occurs (44% above age 25) are higher than those for campus enrolment (26% above age 25). As expected, distance education occurs increasingly frequently over the period examined. Distance education is more evenly spread across birth cohorts, reflecting its usage among older individuals. Working class background is overrepresented in distance compared to campus educational spells (parent manual occupation = 24% for campus and 31% for distance). Distance enrolment occurs more often from small towns and rural areas than do campus studies (campus 11%, distance 28%). Individuals with a migrant background contribute somewhat fewer spells of distance than campus education. Four per cent of campus enrolment spells were contributed by married students, and the corresponding figure for distance enrolment is 8%. Distance enrolment spells are somewhat more common among individuals from the highest income quartile (campus 13%, distance 17%). Distance students spend somewhat more time as students while also having at least 2 years of previous university experience (campus 48%, distance 53%) or having a postgraduate degree (campus 4%, distance 8%). This may possibly simply indicate that skills-upgrade studies are more prominent among distance than among campus courses. Distance enrolment occurs more in the humanities and social sciences than in the natural or technical sciences. Still, health and medicine is a common field of study among distance students. Taken together, this descriptive information indicates that distance studies, more often than campus studies, occur in populations with characteristics that are predictive of a rapid transition to parenthood. This is in line with the general idea that distance study platforms are perceived as being practical from a childbearing perspective and attract individuals with parenthood in mind. At the same time, distance education appears to be a widely dispersed study platform that is also found, for example, among urban (40%) and metropolitan (32%) populations and within normatively expected study ages (e.g. 22–25 = 42%).

**Table 1:** Descriptive statistics

Enrolment status	Subjects	1st parity	Person-months	Share person-months in Uni. enrol.
*Population size and events across all enrolment statuses*				
Not in education	919,924	298,940	42,726,994	
Upper secondary	308,586	2641	2,651,082	
Further	234,213	15,572	4,34,1700	
Full-time University, no distance	307,957	10,962	6,251,307	0.44
Full-time University, distance	40,495	1573	354,294	0.06
Part-time University, no distance	324,413	14,387	4,541,411	0.47
Part-time University, distance	24,326	1157	176,798	0.03
Total^e^	938,768	345,232	61,043,586	100

Variable distribution across campus and distance enrolment	Share person months	
	Campus	Distance
*Age*		
18–21	0.26	0.14
22–25	0.48	0.42
26–29	0.18	0.25
30–33	0.05	0.11
34–37	0.02	0.06
38≥	0.01	0.03
*Period*		
2004	0.12	0.06
2005	0.12	0.09
2006	0.12	0.09
2007	0.12	0.09
2008	0.12	0.10
2009	0.12	0.14
2010	0.12	0.17
2011	0.12	0.18
2012	0.04	0.07
*Cohort*		
1968–1972	0.06	0.12
1973–1977	0.17	0.20
1978–1982	0.24	0.21
1983–1987	0.27	0.24
1988–1990	0.26	0.23
*Social background*		
Manual	0.24	0.31
Non-manual	0.10	0.11
Prof.	0.46	0.41
Self/farm	0.07	0.08
Unknown	0.13	0.10
*Region of residence*		
Metropolitan	0.46	0.32
Urban	0.42	0.40
Small town and rural areas	0.11	0.28
Unknown	0.00	0.00
*Migration status*		
Born in Sweden	0.89	0.93
Born Abroad	0.11	0.07
*Civil status*		
Not married	0.96	0.92
Married	0.04	0.08
Unknown	0.00	0.00
*Income quartile*		
1st	0.24	0.23
2nd	0.36	0.31
3rd	0.27	0.28
4th	0.13	0.17
Unknown	0.00	0.00
*Attained education*		
Compulsory	0.00	0.00
Upper Secondary	0.13	0.10
Post-upper secondary non-tertiary	0.35	0.29
University graduate degree or at least 2 years of studies	0.48	0.53
University postgraduate degree	0.04	0.08
Unknown	0.00	0.00
*Educational field*		
Education and teaching	0.10	0.16
Humanities and art	0.14	0.17
Social sciences	0.33	0.32
Natural sciences	0.11	0.08
Technology	0.07	0.03
Agriculture	0.00	0.00
Health	0.20	0.17
Services	0.04	0.05
Unknown	0.01	0.00
*N* subjects	319,901	53,595
*N* person-months	10,792,718	531,092

### Survival Model Results

Table 2 presents hazard ratios for all enrolment statuses (please see “Appendix” Table 7 for estimates of all included variables). It is clear that, in line with previous research, enrolment has a negative effect on the transition to parenthood. Interestingly, there is a striking difference between the types of educational platforms in tertiary education. The risk of first birth conception during full-time campus enrolment is 70% lower than, or about one-third of, that of someone who is not enrolled (1–0.30). In contrast, the corresponding risk for enrolment that includes a substantial usage of online distance platforms is only 43% lower (1–0.57). For part-time campus enrolment, the risk is 50% lower (1–0.50) and for part-time distance enrolment it is 21% lower (1–0.79). For both full- and part-time enrolment, the negative impact is considerably smaller for periods of enrolment that include a high usage of online distance platforms. Part-time studies are associated with a higher risk than full-time studies, but the positive impact of distance enrolment on the transition to parenthood is seen in both modes of study activity. To put the magnitude of the effect sizes into perspective, the differences in risk between platforms found here are at least as large as the differences between female- and male-dominated educational fields in Norway reported by Lappegård and Rønsen (2005).

**Table 2:** Enrolment effects on first birth conception (hazard ratio estimates and 95% CI)

Not in education	1.00
Upper secondary	0.56*** (0.53–0.58)
Post-upp. sec. non-ter.	0.67*** (0.66–0.68)
Campus full-time	0.30*** (0.29–0.31)
Distance full-time	0.57*** (0.29–0.31)
Campus part-time	0.50*** (0.49–0.51)
Distance part-time	0.79*** (0.75–0.84)
*N*	938,768

Controlled for period, age, age squared, social background, region of residence, migrant status, civil status and educational level

***p < 0.001; **p < 0.01; *p < 0.05

**Fig. 1:**
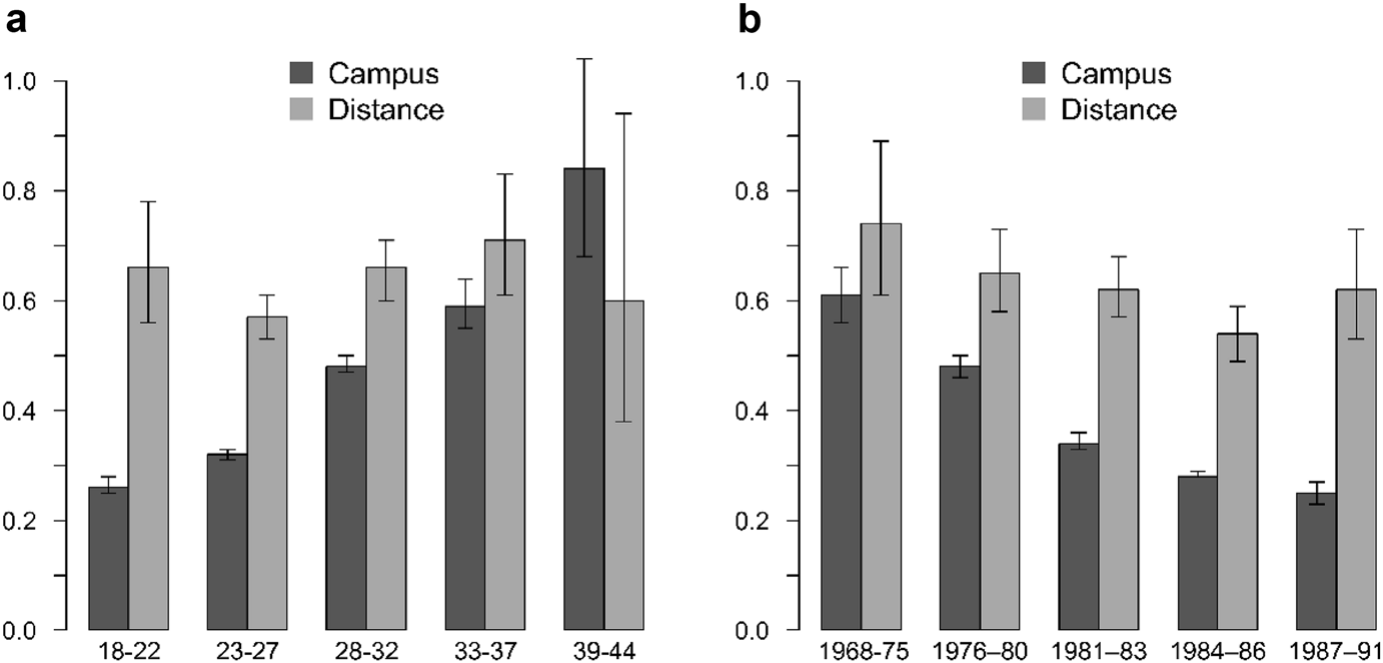
**a** Tertiary enrolment platform effects by age group (hazard ratio estimates and 95% CI). **b** Tertiary enrolment platform effects by cohort group (hazard ratio estimates and 95% CI). *Source*: Swedish Register data. Both figures show estimates controlled for period, age, age squared, social background, region of residence, migrant status, civil status and educational level. ***p < 0.001; **p < 0.01; *p < 0.05

Figure 1a, b presents the estimates for full-time campus and distance enrolment for age and cohort-specific models. The model corresponds to that of Table 2. We see that the gap between distance and campus education decreases, but remains present, for all but the oldest age group. The effect is most pronounced for births at particularly young ages, but the differences also remain clear among the prime first parity age groups of 28–32 years. Confidence intervals overlap for women above the age of 32. For the very late first births, the gap is reversed. The general decrease in the gap across age groups is also consistent with the idea of an overall compression of the childbearing tempo as women approach the biological boundary of childbearing. The analysis based on cohorts shown in Fig. 1b reaffirms the pattern. Later cohorts, who also are younger during the observation period, present the most sizable effect.

### Subgroup Analysis

Some students may consider their main activity to be work rather than studies, and may present corresponding childbearing behaviour. If online platforms are used exclusively to facilitate parallel employment, their impact on non-/sporadically working students should diminish when students are stratified by labour market attachment. Table 3 uses a proxy for such behaviour in the form of quartiles of disposable income. Estimates of upper secondary and further education are excluded for clarity. First, we see that tertiary enrolment has a negative effect on the transition to parenthood irrespective of the level of disposable income. Second, the effects of distance education do not appear to be confounded by an overrepresentation of online platform usage in the highest income group. Rather, in all income groups, distance enrolment is linked to a higher risk of conception than campus enrolment. Finally, while the medium–high and highest income earners have a higher risk of conception across all platforms, the risk associated with distance enrolment increases more clearly across the four income groups.

**Table 3:** Tertiary enrolment effects on first birth conception by income quartiles (hazard ratio estimates and 95% CI)

Not in education	1.00	–
Campus full-time, lowest income quartile	0.30***	(0.29–0.31)
Distance full-time, lowest income quartile	0.38***	(0.34–0.43)
Campus full-time, medium–low income quartile	0.28***	(0.27–0.29)
Distance full-time, medium–low income quartile	0.52***	(0.47–0.56)
Campus full-time, medium–high income quartile	0.29***	(0.28–0.30)
Distance full-time, medium–high income quartile	0.67***	(0.61–0.73)
Campus full-time, highest income quartile	0.43***	(0.41–0.45)
Distance full-time, highest income quartile	0.88*	(0.79–0.98)
Campus part-time, lowest income quartile	0.37***	(0.36–0.38)
Distance part-time, lowest income quartile	0.47***	(0.40–0.54)
Campus part-time, medium–low income quartile	0.43***	(0.41–0.44)
Distance part-time, medium–low income quartile	0.79***	(0.70–0.89)
Campus part-time, medium–high income quartile	0.57***	(0.55–0.59)
Distance part-time, medium–high income quartile	0.86**	(0.78–0.95)
Campus part-time, highest income quartile	0.75***	(0.72–0.77)
Distance part-time, highest income quartile	1.00	(0.90–1.11)
*N*	938,768	

Controlled for period, age, age squared, social background, region of residence, migrant status, civil status and educational level. Subjects with partners of unknown income (*N* = *X*) were grouped in a separate category that is not presented for parsimony. ***p < 0.001; **p < 0.01; *p < 0.05

As is evident from the descriptive data in Table 1, distance enrolment is somewhat more common in the disciplines of education and teaching and health. These are fields that have been argued to attract family-oriented individuals since they, to a greater extent than other disciplines, lead to occupations with lower career penalties for periods away from employment and that offer the possibility of working part-time or flexible hours. Since the strategies, resources and needs of university institutions are discipline specific, institutions differ in their provision of online distance platforms. The link between campus and distance enrolment and fertility may thus be affected by the supply of courses across different disciplines. Table 4 presents enrolment effects by different educational fields. Estimates of upper secondary and further education are excluded for reasons of parsimony. As has been found in previous research (Lappegård and Rønsen 2005), different fields of education are linked to different implications for the transition to parenthood during enrolment. For full-time studies, students of education and teaching and health stand out as having a high risk, while students of technology, natural science, social science and agriculture are quite unlikely to become parents during enrolment. Students of services, humanities and arts and unknown fields of study lie between these two groups. These differences across different fields could reflect human capital investment in prospective careers, such that students of technology anticipate a greater need to establish themselves on the labour market than aspiring teachers or nurses. The differences might also reflect a selection of individuals who are looking to have children into educational fields that lead to so-called family-friendly occupations. The nature of the studies in different fields may also be more or less compatible with the transition to parenthood. All fields carry a higher risk in part-time enrolment than in full-time enrolment, and teaching students who are studying part-time show a particularly high risk. As regards the differences across platform, distance enrolment has consistently weaker effects than campus enrolment in the corresponding educational fields. Overall, it appears that the effect of distance education is not driven by distance course supply in specific educational fields. Among part-time distance enrolees in education and teaching, the risk of making the transition to parenthood is not significantly different from that of individuals who are not enrolled in education, but these estimates also have fairly wide confidence intervals. However, the risk of making the transition to parenthood also approaches that of the non-enrolled population for those in full-time distance enrolment in teaching and education (CI 0.80–0.96). These findings could be driven by unknown qualitative aspects of distance education in certain fields. It might, for example, be that distance skills-upgrade courses are more commonly offered within teaching and education, and are being taken by students who are already rooted in a profession, and who are at higher risk of becoming parents. However, the findings may also reflect a double selection effect whereby individuals who are looking to have children are selecting into both family-friendly fields and, as I have argued, family-friendly educational platforms. In this case, one interpretation would be that students of teaching are using distance education as one part of their strategy to achieve their fertility intentions during their period of study.

**Table 4:** Tertiary enrolment effects by educational field on first birth conception rates (hazard ratio estimates and 95% CI)

Not in education	1	–
Campus full-time, education and teaching	0.51***	(0.48–0.53)
Distance full-time, education and teaching	0.88**	(0.80–0.96)
Campus full-time, humanities and art	0.29***	(0.28–0.31)
Distance full-time, humanities and art	0.39***	(0.34–0.45)
Campus full-time, social sciences	0.24***	(0.23–0.25)
Distance full-time, social sciences	0.42***	(0.38–0.47)
Campus full-time, natural sciences	0.20***	(0.18–0.22)
Distance full-time, natural sciences	0.48***	(0.40–0.58)
Campus full-time, technology	0.16***	(0.14–0.17)
Distance full-time, technology	0.24***	(0.15–0.38)
Campus full-time, agriculture	0.20***	(0.15–0.27)
Distance full-time, agriculture	0.00***	(0.00–0.00)
Campus full-time, health	0.39***	(0.38–0.40)
Distance full-time, health	0.77***	(0.69–0.85)
Campus full-time, services	0.25***	(0.23–0.28)
Distance full-time, services	0.55***	(0.45–0.68)
Campus full-time, unknown	0.21***	(0.17–0.25)
Distance full-time, unknown	0.60	(0.31–1.14)
Campus part-time, education and teaching	0.79***	(0.76–0.82)
Distance part-time, education and teaching	1.11	(0.99–1.25)
Campus part-time, humanities and art	0.43***	(0.41–0.45)
Distance part-time, humanities and art	0.59***	(0.50–0.69)
Campus part-time, social sciences	0.41***	(0.40–0.42)
Distance part-time, social sciences	0.64***	(0.57–0.72)
Campus part-time, natural sciences	0.40***	(0.37–0.42)
Distance part-time, natural sciences	0.80*	(0.66–0.98)
Campus part-time, technology	0.35***	(0.32–0.38)
Distance part-time, technology	0.94	(0.67–1.30)
Campus part-time, agriculture	0.28***	(0.20–0.39)
Distance part-time, agriculture	0.34	(0.05–2.39)
Campus part-time, health	0.64***	(0.61–0.66)
Distance part-time, health	0.89	(0.78–1.01)
Campus part-time, services	0.47***	(0.43–0.52)
Distance part-time, services	0.81	(0.62–1.06)
Campus part-time, unknown	0.40***	(0.32–0.50)
Distance part-time, unknown	2.00*	(1.02–3.91)
*N*	938,768	

Controlled for period, age, age squared, social background, region of residence, migrant status, civil status and educational level. ***p < 0.001; **p < 0.01; *p < 0.05

### Sensitivity Analyses and Alternative Specifications

As discussed in the methods section, the separation of enrolment terms into campus and distance categories is based on a specific definition. In the absence of a gold-standard, I have reasoned that above half a term worth of credits constitutes a plausible threshold for a substantial usage of online platforms. To examine the validity of this threshold, I modelled increasing categorical levels of the amount of distance study. I expect there to be a certain substantive amount of ODE usage needed to make a difference for demographic behaviour. A greater proportion of distance enrolment should have a more salient effect than smaller proportions. If there is an effect of some usage of online distance platforms, but this is similar across different amounts of distance education during a given term, then it is likely that the results are being driven by forces with little relevance to my argument. Model 1 in Table 5 presents the main effects of tertiary enrolment categories on the risk of first birth conception, by differing amounts of distance usage. Non-enrolment is the reference category. Students taking for example 7.5 ETCS (one standard course) online during a term show no or only minor differences by comparison with students enrolled in campus only (0.29 vs. 0.30). Students taking 8–15 distance credits show only a marginal difference (0.35 vs. 0.30) with overlapping confidence intervals. Noticeable higher estimates are found for higher amounts (above 15 ETCS) of online education (0.52 vs. 0.30 and 0.62 vs. 0.30). This suggests an association between distance education and student fertility, and also strengthens the argument for the use of a threshold (of around at least half a term worth of credits) for substantial online usage. To provide an alternative perspective, Model 2 instead specifies full-time enrolment (in any platform) as the reference category and includes a continuous variable for online ETCS credits to produce a linear estimation (capped at 30 ETCS, mean = 13.1, SD = 8.3). This estimate suggests that the risk of becoming a parent increases by 2.6 percentage points for each additional online credit point acquired within a term of full-time education, relative to a full-time exclusively on-campus term.

**Table 5:** Full-time tertiary enrolment effects on first birth conception across amount of online education in full-time enrolment (hazard ratio estimates and 95% CI)

	Model 1	Model 2
Not in education	1.00	3.34 (3.29–3.42)
Full-time campus	0.30*** (0.29–0.31)	1.00
Full-time distance (1–7 distance ETCS)	0.29*** (0.26–0.33)	–
Full-time distance (8–14 distance ETCS)	0.35*** (0.27–0.44)	–
Full-time distance (15–22 distance ETCS)	0.52*** (0.49–0.56)	–
Full-time distance (> 22 distance ETCS)	0.62*** (0.58–0.67)	–
Distance ECTS (continuous)	–	1.02*** (1.02–1.02)
*N*	938,768	938,768

Including controls for period, age, age squared, social background, region of residence, migrant status, civil status and educational level

***p < 0.001; **p < 0.01; *p < 0.05

Several alternative sensitivity tests to the main findings presented in Table 2 are displayed in Table 6. For reference, the results of Table 2 are shown again in Model 1. The interpretations following Model 1 have presumed that distance and campus students have somewhat similar study trajectories. Even if studies constitute the main activity during a given term, it might be speculated that online distance education might be disproportionately used by students with no intention of pursuing a complete programme of formal higher education. Studying with a more leisurely orientation could in itself reduce the effect of enrolment on childbearing behaviours, which would make comparisons with campus studies moot. In Model 2, individuals who did not receive a degree are excluded from the student population. Compared to Model 1, the risk of first birth conception during enrolment becomes lower, but the gap between distance and campus enrolment persists. Another way in which distance and campus studies may be functionally different would be if ODE is overrepresented in the context of sporadic non-continuous studies. Anticipatory behaviour might also lead individuals who are opting out of the labour market in expectation of becoming pregnant to resort to short-term enrolment spells as a temporary activity. In Model 3, I include a dummy for single-spell terms, defined as an enrolment term that is not sequentially preceded by a previous enrolment term. We again see a reduction in the risk of first birth conception in tertiary enrolment. It is therefore plausible that the effects of platform type can in part be attributed to specific types of study strategies. However, the estimates presented in Models 2 and 3 still reproduce the overall patterns of the substantive differences between platforms found in Model 1. This result hints that differences between online and campus enrolment in the transition to adulthood are not a direct artefact of platform-specific educational trajectories.^2^

**Table 6:** Enrolment effects on first birth conception and first birth (hazard ratio estimates and 95% CI). Alternative specifications

	Model 1^a^	Model 2^b^	Model 3^c^	Model 4^d^	Model 5^e^
Not in education	Ref.	Ref.	Ref.	Ref.	
Upper secondary	0.56*** (0.53–0.58)	0.55*** (0.52–0.57)	0.54*** (0.52–0.56)	0.20*** (0.19–0.22)	0.57*** (0.55–0.60)
Post-upp. sec. non-ter.	0.67*** (0.66–0.68)	0.71*** (0.70–0.72)	0.67*** (0.65–0.67)	0.31*** (0.30–0.32)	0.68*** (0.67–0.69)
Campus full-time	0.30*** (0.29–0.31)	0.18*** (0.17–0.19)	0.25*** (0.24–0.25)	0.13*** (0.13–0.13)	0.31*** (0.31–0.32)
Distance full-time	0.57*** (0.29–0.31)	0.41*** (0.38–0.46)	0.44*** (0.42–0.46)	0.32*** (0.30–0.34)	0.58*** (0.54–0.61)
Campus part-time	0.50*** (0.49–0.51)	0.34*** (0.32–0.35)	0.36*** (0.35–0.37)	0.34*** (0.34–0.35)	0.52*** (0.51–0.53)
Distance part-time	0.79*** (0.75–0.84)	0.68*** (0.60–0.77)	0.47*** (0.45–0.50)	0.52*** (0.48–0.55)	0.86*** (0.80–0.92)
*N*	938,768	651,831	938,768	927,159	938,768

^a^Main model

^b^Conditional on enrolment resulting in degree

^c^Controlling for isolated study spells

^d^Date of birth instead of date of conception. Controlled for period, age, age squared, social background, region of residence, migrant status, civil status and educational level

^e^Latest year of studies treated as non-enrolment

***p < 0.001; **p < 0.01; *p < 0.05

I have used date of conception to ameliorate state/date issues since childbirth might increase the likelihood of anticipatory changes in educational status (Liefbroer and Corijn 1999). However, if most conceptions occur near the completion of tertiary studies, birth will occur following the termination of enrolment and does not, arguably, constitute student fertility. If such behaviour is overrepresented among the users of online platforms (i.e. if distance studies are disproportionately used during the very final stages of university studies) using the date of conception will produce artificial differences. Model 4 presents estimates of first birth risk using date of birth rather than conception. Across all enrolment types, the hazard of student parenthood is consistently lower compared to the estimates in Model 1, indicating that among students, the transition to parenthood is skewed towards later periods of the enrolment period. However, the pattern of differences between platforms remains, both for full- and part-time studies. To further tap into this issue, Model 5 uses date of conception but treats the last two terms of studies as non-enrolment. This specification counts only events occurring within study spells “mid-term” as student childbearing. Again, campus distance differences are smaller compared to Model 1 but a substantive gradient remains. This suggests that online platforms are compatible with the student transition to parenthood throughout the period of tertiary studies, and are not solely utilized to ease the transition towards the very end of the study period.

As argued in the theoretical section, I have assumed that the relationship between distance education and childbirth could go both ways. Thus far, the survival models censor on the event of childbirth, and they hold non-enrolment rather than campus enrolment as the comparison. I include complementary analyses of how childbearing and conception predict distance enrolment, holding campus enrolment as the reference category. “Appendix” Table 8 predicts platform type in a cross section of the entire female student population in 2004 and 2011. Compared to campus studies, distance studies are slightly favoured among those who already are parents, but clearly favoured during first conception periods. This reflects the reasonable conclusion that ODE may be preferred by parents in general, and that its effect is not only confined to impacting choices regarding transition to parenthood.

Finally, in “Appendix” Tables 9 and 10, I look at second-order parity. If distance education impacts on higher parity student childbearing, it is again support for the practicality of ODE. However, we see in Table 9 that platform differences are smaller here than in first-parity models. This may indicate that higher-order student parents are highly select, but runs counter to the intuitive explanation of mothers using ODE to combine multiple roles and activities. Estimating second parity rather than transition into parenthood has the advantage of identifying partners via biological ties to firstborns. I use this information to show differences in distance enrolment over partner’s income quartiles. “Appendix” Table 10 shows that higher partner income is associated with a higher likelihood of student childbearing, and that this pattern is quite similar for campus and distance students.

## Discussion and Conclusion

Educational enrolment is pivotal to understanding fertility postponement. Further, large student stocks and increasing ages at enrolment and at the completion of education give cause to explore the antecedents of student fertility. The present study has extended the literature on the heterogeneous effects of education on fertility by considering the difference between online and on-campus education. Using register data on all Swedish women born from 1968 to 1991, I have assessed whether the risk of transition to parenthood during university studies is related to the usage of online distance education (ODE).

The results show that when all or a high proportion of a period of enrolment is conducted at distance, the negative association between educational enrolment and the risk of transition to parenthood is considerably smaller. Descriptive findings show that online studies are disproportionately utilized by individuals with a high transition risk, such as older and married students. At the same time, a higher likelihood of first birth is still found among distance students across age and cohort groups, in so-called family-friendly educational fields and among students within different income brackets even when controls are included for sociodemographic characteristics. I have shown that while study patterns—the way individuals use ODE and brick-and-mortar education, respectively—probably explain part of the effect, the impact of ODE on fertility is found for full-time and part-time students, for students who do and do not graduate and for students with both adjacent study spells and with no contiguous study spells.

I have suggested a few reasons for the observed association. Some of the fundamental attributes of online distance learning, such as flexibility, mobility and independence from campus, are consistent with the needs of parent students. Distance education may enable fertility by promoting double-status positions (Spéder and Bartus 2017) and by transforming studies into a domestic activity that is compatible with work and parenthood. Such an interpretation is supported by the fact that effects were particularly prominent among part-time students, students with higher yearly incomes, student earners and students aiming at family-oriented occupations such as teaching. The property of enabling fertility need not imply a causal relationship, but it does provide an intuitive explanation for why distance education would be a preferred option in connection with transitioning into parenthood. The central take-home message from the present study is that the educational platform may be considered an important factor, with differentiated effects on fertility. This finding is of particular importance in light of the rapid expansion of distance education.

Will the diffusion of online platforms causally increase student fertility or the fertility tempo among those with a tertiary education? The present paper does not employ the type of design that would be necessary to answer this question. Many of the students who become parents during distance studies are probably a select group who would have become parents regardless of the available study platforms. In this case, the diffusion of online platforms produces a dividend, based on educational platform, for an existing demographic behaviour, but would not impact on student fertility as such. Indeed, fertility intentions may govern the choice into or type of tertiary education (Cohen et al. 2011). Yet another counterfactual is that individuals observed to be taking online courses, and who experience childbirth during their studies, might have opted out of tertiary education altogether if ODE had not been available. However, studies in family planning (Hammerslough 1992), for example, have shown that fertility and family behaviour are impacted by access to resources and innovations that provide practical assistance to intended behaviours. To the extent that distance education provides enabling practical assistance to student parenthood, it is conceivable that at least a part of the student population are using online platforms as a tool to help them realize their childbearing intentions. This group might be responsive to the increasing supply of distance online courses. The results in the present paper can thus be seen as providing a tentative indication of the possibility that online distance education reduces the postponement of childbearing for subgroups of university-educated individuals, increasing their chances of realizing their fertility intentions.

Online distance studies can be conceptualized as a technology that enables particular fertility behaviours. The different rates of transition to parenthood during enrolment hint that campus and distance education may be qualitatively different experiences. One possible interpretation is that as online distance education becomes more ubiquitous within tertiary educational institutions, the university experience changes; campus life becomes increasingly optional, flexibility increases and distance and commuting become less of a problem, providing a leeway for student parenthood. This is a plausible scenario that constitutes a clear illustration of how the life course is regulated by concrete structuring environments such as university campuses, and of how technological innovations can alter these structuring conditions. This illuminates the need for further study of ODE as a means by which information communication technologies (ICT) are affecting the life course (Chesley and Johnson 2014).

Finally, the awareness of a relationship between the transition to parenthood and distance education might be informative for policy makers. Caution is needed on this point. This is the case in part because the present study is not based on a causal design, but also because education functions within broader complex systems whose effects are difficult to forecast. Nonetheless, actors with an interest in childbearing or lifelong learning policies might benefit from a careful consideration of the findings from this study.

Several limitations should be noted. While I distinguish study intensity, this study cannot address the issue that the nature of online studies may be different from that of campus studies. This also includes the issue of the financial returns to education, which is fundamentally tied to fertility. Better data on the content of online and campus education, including a comparison of complete programmes of tertiary studies, would have been of benefit here. Furthermore, individuals were observed during a limited 8-year period and no data have been included from the period after 2012. This is a period in which new technology has powerfully expanded the use of online learning platforms. The latest enrolment cohorts on which this study is based, and which contain the highest proportions of distance students, completed their education subsequent to the conclusion of the observation period. A longer time frame would be beneficial, but this is of course a shortcoming that affects most studies of current phenomena. Finally, while the population level coverage is advantageous with respect to external validity, Sweden may also be a problematic case to generalize from, since higher education and parenthood occur in a context of subsidized students loans and parental leave schemes that are not representative of all developed countries.

Despite these limitations, the present study has made several contributions. It is the first paper to investigate the role of online distance tertiary education in the transition to parenthood. I draw on standard theories of transition to parenthood to elaborate on the function of distance education for childbearing postponement via student parenthood. I provide novel population-based empirical findings that provide support for the existence of a link between distance education and the transition to parenthood across several relevant subgroups. The present paper has taken a first step that will provide a basis for future inquiries into the possible role of distance education for childbearing behaviour. Addressing selectivity and reverse causality is important, and should be an aim for future studies. Identifying a random components in regional Internet access, recent studies use broadband rollout to tease out exogenous variation in ICT usage (see Bellou 2015; Billari, Giuntella and Stella 2017). In the present case, broadband rollout is highly diffused during the observed period, and is linked to distances to university and other predictors of enrolment, which complicates its function as an instrument. However, for other contexts and periods, this strategy, as a means of estimating outcomes of ODE net of selection, holds great promise. Furthermore, despite the extensive coverage of the data in the present study, turning to limited but more detailed sources is likely to be fruitful. Having in-depth information on ODE and brick-and-mortar versions of similar educational programs, or on particular tertiary institutions that implement online learning platforms at different periods in time, may allow for different designs that might aid inference. On this note, one important question is whether or not the spread of online education enables non-traditional students to enrol and thus causally empowers underprivileged groups. The flow into online education among those who are already parents, and its impact on higher-order births, is also a topic for future research. Much can be done to develop and model mechanisms. For example, do distance students’ patterns of social interaction, partnering and labour market trajectories differ from those of their peers on campus, and does this affect fertility? Also, given the well-documented role of the university as a marriage market, what are the implications of online distance enrolment for assortative partnership formation? Examining large educational institutions that offer full programs on both online and campus platforms might provide a means of addressing these issues.

## Appendix

See Tables 7, 8, 9 and 10.

**Table 7:** Effects of all covariates included in the model from Table 2 on first birth conception (hazard ratio estimates and 95% CI)

*Enrolment status*		
Not in education (ref)	1.00	–
Upper secondary	0.56***	(0.53–0.58)
Post-upper secondary non-tertiary	0.67***	(0.66–0.68)
Campus full-time	0.30***	(0.29–0.31)
Distance full-time	0.57***	(0.54–0.60)
Campus part-time	0.50***	(0.49–0.51)
Distance part-time	0.79***	(0.75–0.84)
*Period*		
2004 (ref)	1.00	–
2005	1.00	(0.99–1.02)
2006	0.99	(0.98–1.00)
2007	1.00	(0.99–1.01)
2008	1.01	(0.99–1.02)
2009	1.02**	(1.01–1.04)
2010	0.98**	(0.97–0.99)
2011	0.97***	(0.96–0.99)
2012	0.69***	(0.67–0.70)
*Social background*		
Manual labour (ref)	1.00	–
Non-manual labour	0.94***	(0.93–0.95)
Professional	0.85***	(0.84–0.86)
Self-employed or farmer	0.90***	(0.89–0.92)
Unknown	0.94***	(0.93–0.96)
*Region of residence*		
Metropolitan (ref)	1.00	–
Urban	1.13***	(1.12–1.13)
Small town and rural areas	1.27***	(1.26–1.28)
Unknown	0.66***	(0.62–0.70)
*Migration status*		
Born in Sweden (ref)	1.00	–
Born Abroad	0.82***	(0.81–0.83)
*Civil status*		
Not married (ref)	1.00	–
Married	4.12***	(4.08–4.15)
Unknown	1.00	(1.00–1.00)
*Attained education*		
Compulsory (ref)	1.00	–
Upper secondary	0.85***	(0.84–0.87)
Post-upper secondary non-tertiary	0.81***	(0.79–0.82)
University graduate degree or at least 2 years of studies	1.10***	(1.08–1.11)
University postgraduate degree	1.13***	(1.12–1.15)
Unknown	0.53***	(0.51–0.55)
*Age*	2.24***	(2.22–2.26)
*Age squared*	0.99***	(0.99–0.99)
*N*	938,768	

****p* < 0.001; ***p* < 0.01; **p* < 0.05

**Table 8:** Odds of enrolment in distance education compared to campus education, by parity, conception and control variables. Cross section of student population in 2004 and 2011 (odds ratios and 95% CI)

	2004		2011	
*Parity*				
0 (ref)	1.00	–	1.00	–
1	1.32***	(1.25–1.38)	1.11***	(1.05–1.18)
2	1.19***	(1.14–1.25)	1.06*	(1.01–1.12)
3 or more	1.13***	(1.06–1.19)	0.98	(0.92–1.04)
*Conception* ^a^				
No birth	1.00	–	1.00	–
1st birth	1.17***	(1.07–1.29)	1.62***	(1.47–1.80)
2nd birth	1.43***	(1.28–1.59)	1.54***	(1.35–1.76)
3rf birth	1.07	(0.89–1.28)	1.16	(0.95–1.42)
*Social background*				
Manual labour (ref)	1.00	–	1.00	–
Non-manual labour	1.60***	(1.48–1.73)	1.29***	(1.16–1.44)
Professional	1.52***	(1.40–1.65)	1.33***	(1.19–1.48)
Self-employed or farmer	1.37***	(1.26–1.49)	1.37***	(1.22–1.53)
Unknown	1.57***	(1.43–1.72)	1.30***	(1.15–1.46)
*Region of residence*				
Metropolitan (ref)	1.00	–	1.00	–
Urban	1.29***	(1.25–1.33)	1.38***	(1.33–1.43)
Small town and rural areas	2.49***	(2.40–2.58)	3.00***	(2.89–3.13)
Unknown	2.29**	(1.25–4.22)	9.34***	(5.31–16.43)
*Migration status*				
Born Abroad	0.83***	(0.78–0.88)	0.82***	(0.75–0.89)
*Civil status*				
Married	1.20***	(1.16–1.25)	1.18***	(1.14–1.23)
*Attained education*				
Compulsory (ref)	1.00	–	1.00	–
Upper secondary	1.49	(0.95–2.33)	0.80	(0.61–1.05)
Post-upper secondary non-tertiary	0.80	(0.51–1.25)	0.56***	(0.43–0.74)
University graduate degree	0.71	(0.45–1.11)	0.80	(0.61–1.05)
University postgraduate degree	1.08	(0.51–2.29)	0.54*	(0.29–0.98)
*Age*	1.37***	(1.35–1.39)	1.35***	(1.33–1.37)
*Age square*	1.00***	(1.00–1.00)	1.00***	(1.00–1.00)
*N*	186,708		178,311	

^a^Birth occurred during 2005/2012

**Table 9:** Tertiary enrolment effects on second birth conception (hazard ratio estimates and 95% CI)

Not in education	1.00
Upper secondary	0.56*** (0.49–0.63)
Post-upp. sec. non-ter.	0.76*** (0.75–0.77)
Campus full-time	0.62*** (0.60–0.63)
Distance full-time	0.70*** (0.65–0.74)
Campus part-time	0.76*** (0.74–0.78)
Distance part-time	0.86*** (0.80–0.92)
*N*	938,768

Controlled for period, age, age squared, social background, region of residence, migrant status, civil status and educational level

****p* < 0.001; ***p* < 0.01; **p* < 0.05

**Table 10:** Tertiary enrolment effects on second birth conception by partners’ income quartiles (hazard ratio estimates and 95% CI)

Not in education	1.00	–
Campus full-time, *p* lowest income quartile	0.46***	(0.43–0.50)
Distance full-time, *p* lowest income quartile	0.53***	(0.44–0.64)
Campus full-time, *p* medium–low income quartile	0.59***	(0.56–0.62)
Distance full-time, *p* medium–low income quartile	0.69***	(0.61–0.77)
Campus full-time, *p* medium–high income quartile	0.71***	(0.67–0.74)
Distance full-time, *p* medium–high income quartile	0.74***	(0.66–0.83)
Campus full-time, *p* highest income quartile	0.79***	(0.74–0.83)
Distance full-time, *p* highest income quartile	0.83**	(0.73–0.95)
Campus part-time, *p* lowest income quartile	0.57***	(0.54–0.61)
Distance part-time, *p* lowest income quartile	0.81*	(0.67–0.98)
Campus part-time, *p* medium–low income quartile	0.72***	(0.68–0.75)
Distance part-time, *p* medium–low income quartile	0.81**	(0.72–0.93)
Campus part-time, *p* medium–high income quartile	0.83***	(0.80–0.87)
Distance part-time, *p* medium–high income quartile	0.92	(0.81–1.04)
Campus part-time, *p* highest income quartile	0.94**	(0.90–0.98)
Distance part-time, *p* highest income quartile	0.90	(0.78–1.04)
*N*	477,323	

Controlled for period, age, age squared, social background, region of residence, migrant status, civil status and educational level and income rank. Second-birth with first-birth parents: Censoring at divorce

****p* < 0.001; ***p* < 0.01; **p* < 0.05

## Footnotes

1 In practice, this means that half-time distance studies range from 15 to 22 while half-time campus studies range from 1 to 22. I decided to keep the present imbalance, as the main purpose is to discriminate between part-time and full-time enrolment. Limiting part-time students to a 15–22.5 credits definition did not impact on the estimates.

2 These estimates must be interpreted with some caution due to the problems associated with anticipatory analysis.
